# Undifferentiated pleomorphic sarcoma of the thyroid: A case report and ƚiterature review

**DOI:** 10.1002/ccr3.2710

**Published:** 2020-02-11

**Authors:** Van Bang Nguyen, Van Vy Hau Nguyen, Binh Thang Tran, Chi Van Le, Cong Thuan Dang, Jung Hwan Baek

**Affiliations:** ^1^ Center of Endocrinology and Diabetes Family Hospital Da Nang Vietnam; ^2^ Department of Cancer Control and Population Health National Cancer Center Graduate School of Cancer Science and Policy 323 Ilsan Goyang South Korea; ^3^ Internal Medicine Department Hue University of Medicine and Pharmacy Hue University Hue city Vietnam; ^4^ Department of Pathology Hue University of Medicine and Pharmacy Hue University Hue city Vietnam; ^5^ Department of Radiology and Research Institute of Radiology University of Ulsan College of Medicine Asan Medical Center Seoul Korea

**Keywords:** malignant fibrous histiocytoma, sarcoma, thyroid gland, undifferentiated pleomorphic sarcoma

## Abstract

This report describes a patient who presented with a large thyroid nodule and compressive symptoms. Immunohistochemical staining showed diffuse marked reactivity for vimentin and focal reactivity for CD68 and Ki‐67 that is compatible with primary undifferentiated pleomorphic sarcoma of the thyroid. This report emphasizes and discusses extremely rare thyroid cancer type.

## Graphical abstract

Primary thyroid undifferentiated pleomorphic sarcoma is fast‐growing neck tumor in the elderly patients clinically. Although all routine tests before thyroid radiofrequency ablation (RFA) show benign thyroid nodule, we chose surgery instead of RFA.

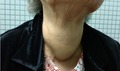

## 1 INTRODUCTION

Sarcomas are a rare and heterogeneous group of malignant tumors of mesenchymal origin that comprise less than one percent of all adult malignancies and 12 percent of cancers in children.^1^, ^2^, ^3^ Approximately 80 percent of new cases of sarcoma develop from soft tissue.^3^ Histologically, the diagnosis of a soft tissue sarcoma is made on the basis of morphologic patterns. Immunohistochemical staining often aids in the identification of the probable tissue of origin.

Malignant fibrous histiocytoma (MFH), completely renamed “undifferentiated pleomorphic sarcoma” (UPS) in the 2013 World Health Organization classification of soft tissue sarcoma, is the most uncommon subtype of soft tissue sarcoma.^4^ Primary UPS of the thyroid is a subtype of primary thyroid sarcoma (reported frequency ranges from 0.01% to 1.5%), which extremely rarely occurs in the thyroid gland.^5^, ^6^, ^7^ To the best of our knowledge, there are only a few well‐documented primary UPS of the thyroid in the literature and this is the first report in Vietnam. In this report, we describe a 79‐year‐old woman with primary UPS of the thyroid gland and review the literature.

## 2 CASE PRESENTATION

A 79‐year‐old woman presented with a fast‐growing, painless thyroid nodule and symptoms of dysphagia and dysphonia for six months. She reported a history of hypertension, no history of radiation to the neck, and no family history of any malignancy. She was admitted to hospital for a tentative thyroid radiofrequency ablation (RFA).

**Figure 1:**
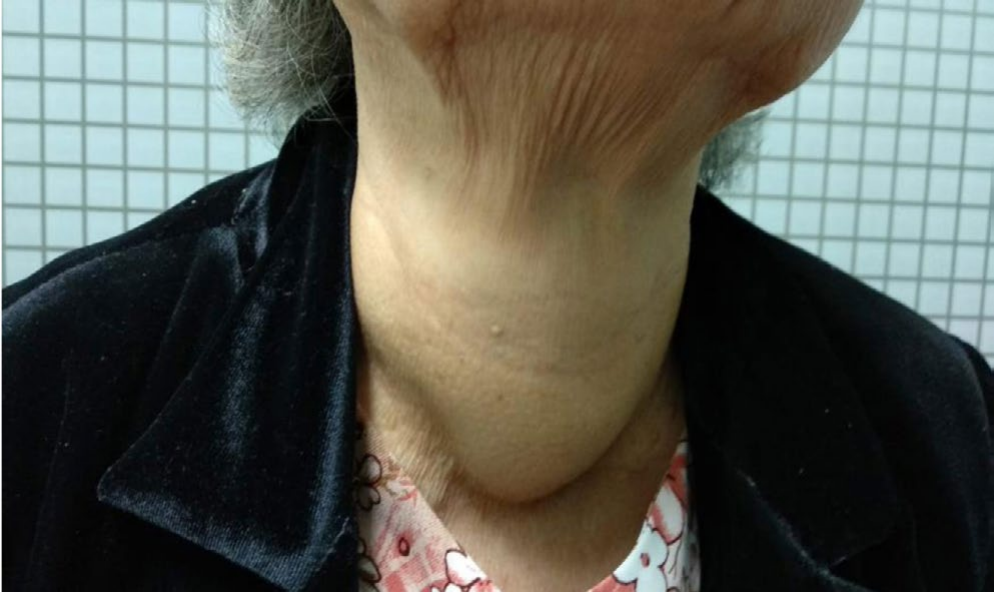
A 79‐y‐old woman visited our hospital because of a fast‐growing neck mass

**Figure 2:**
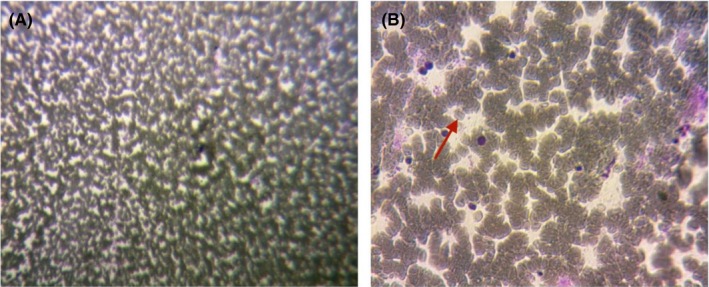
FNA of thyroid mass showed a hemorrhagic aspirate (red arrow)

**Figure 3:**
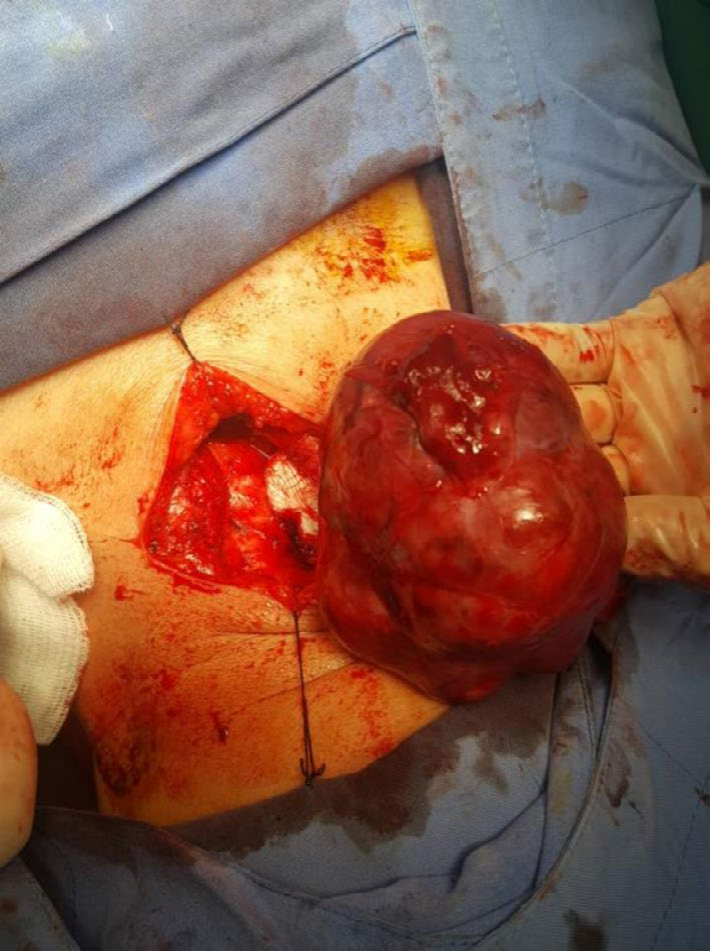
Total thyroid gland removed

On physical examination, a very large, solid, and irregular thyroid nodule was detected (Figure 1). No lymph node was found in the lateral neck. Thyroid function tests (FT4, thyrotropin) and other tests (CBC, liver, and renal function tests) were normal with serum FT4 1.18 ng/dL (normal range [NR] 0.93‐2.22); thyrotropin 0.59 microU/mL (NR 0.27‐4.2). Her chest X‐ray showed no abnormal findings. The thyroid ultrasonography showed a 45 × 32 mm mixed echogenicity mass which occupied nearly the entire thyroid gland, with no calcification, no vascularity, and no lymph node involvement. Fine Needle Aspiration (FNA) of the thyroid mass yielded hemorrhagic aspirate (Figure 2A,B) with diagnosis of a large benign thyroid nodule. The patient refused a core needle biopsy. A total thyroidectomy instead of RFA was performed because of compressive symptoms and high risk of malignancy (Figure 3).

**Figure 4:**
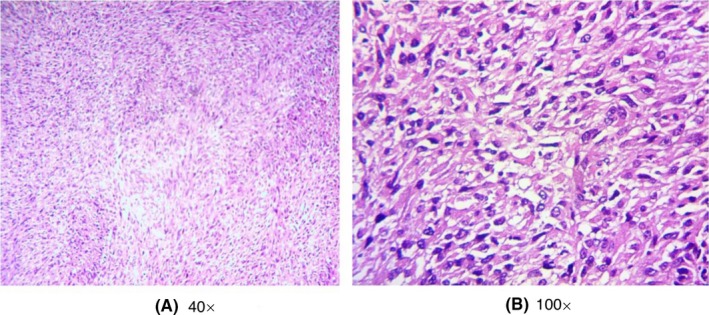
Moderately differentiated thyroid sarcoma on histopathology

**Figure 5:**
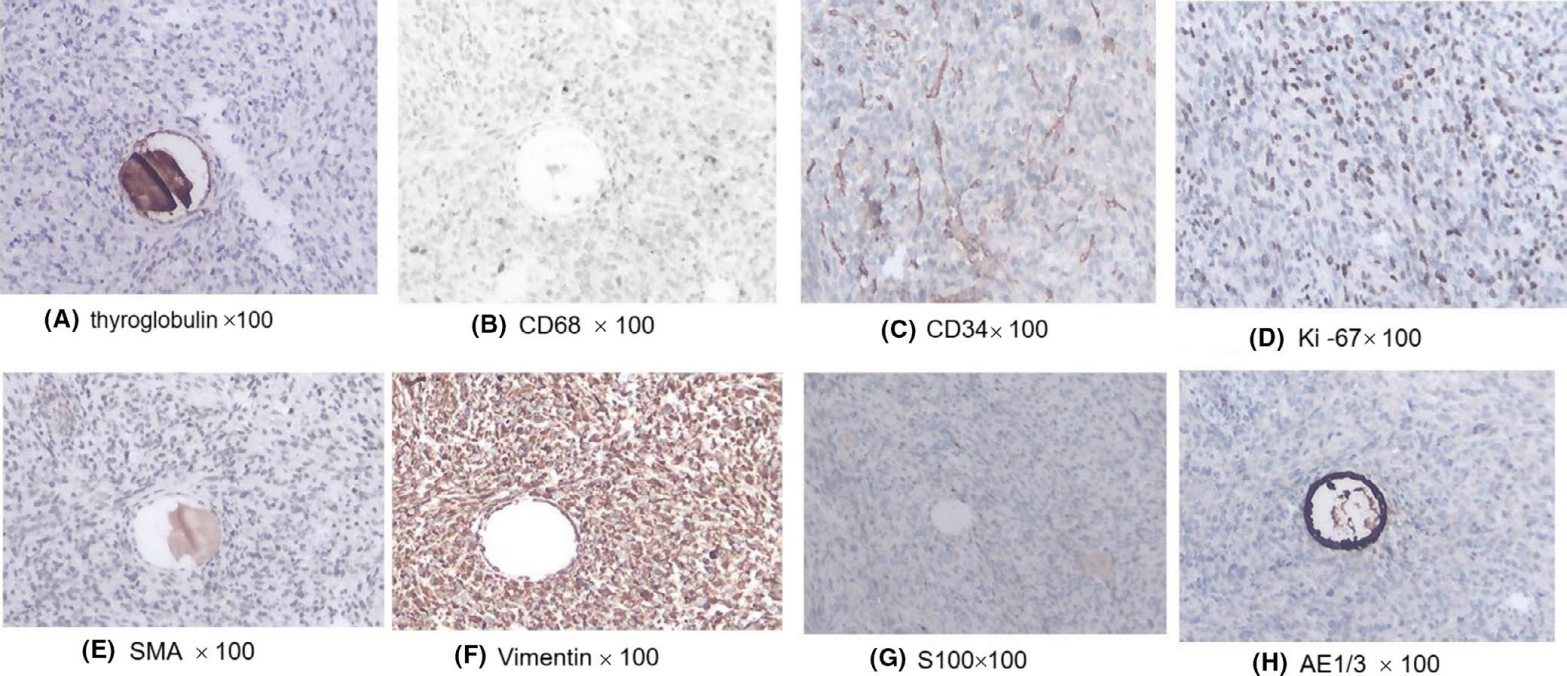
Results of immunohistochemical staining

Histopathologic examination (HE stain) revealed the tumor cells were spindle‐shaped, round, or ovoid, and arranged in a whirlpool fashion with invasive growth, with high mitotic activity and areas of necrosis. It is consistent with a moderately differentiated thyroid sarcoma (Figure 4A,B). Immunohistochemical staining showed diffuse marked reactivity for vimentin and focal reactivity for CD68 and Ki‐67 (positive 20%). No reactivity for AE1/3, thyroglobulin, S100, or smooth muscle actin was detected (Figure 5A‐H). Histopathologic and immunohistochemical staining features were suggestive of UPS of the thyroid. No postoperative chemo‐ or radiotherapy was given because her daughter refused. The whole‐body scan detected no metastasis to distant organs and lymph nodes. After 6 months of follow‐up, local recurrence was detected and no further treatment was undertaken because of her poor performance status.

## 3 DISCUSSION AND CONCLUSIONS

There are four major types of thyroid cancer. The most common types are papillary thyroid cancer—85% and follicular thyroid cancer—10%. Less frequent types are thyroid Hurthle cell carcinoma and medullary carcinoma. Anaplastic type is quite rare, only 1%‐2%.^8^ Primary thyroid lymphoma (PTL) make up from 0.5% to 5.0% of all thyroid malignancies.^9^, ^10^ Primary thyroid sarcoma (PTS) is very rare with a reported incidence ranging from 0.01% to 1.5%. UPS of the thyroid is a subtype of thyroid sarcoma which is extremely rare with 20 cases reported in the English language literature, and only three cases reported in the past ten years.

Definitive diagnosis is crucial in determining prognosis treatment strategy. UPS, PTL, and anaplastic thyroid cancer are present as rapidly growing thyroid tumors in elderly patients. Unfortunately, ultrasonographic features and FNA cytology may not help to differentiate these entities. When diagnostic surgery is not available, core needle biopsy will help to exclude PTL from UPS and anaplastic thyroid cancer which characterized by diffuse infiltration of lymphoid cells.^10^, ^11^ To distinguish thyroid UPS from anaplastic thyroid carcinoma, histopathologic features and immunohistochemical stains are critical. Both of them show the characteristic spindle cell, pleomorphism, and storiform pattern which distinguish them from nonmesenchymal thyroid cancers. Immunohistochemically, both of them are positive for vimentin but only thyroid UPS is positive for α1‐antitrypsin and CD68, which enable definite diagnosis (Table 1).

**Table 1:** Histopathologic features and immunohistochemical results in thyroid UPS, anaplastic thyroid carcinoma, and thyroid lymphoma

Rare thyroid cancer	Histopathologic features	Immunohistochemistry				
		Vimentin	CD68	CK	α1‐antitrypsin	LCA
UPS‐Thyroid	Spindle cell, pleomorphism, storiform pattern	+	+	–	+	‐
Anaplastic carcinoma of thyroid		+	‐	+	‐	‐
Primary thyroid lymphoma	A diffuse infiltration by T or B lymphocytic cells	‐	‐	‐	‐	+

Abbreviations: (‐), negative; (+), positive; ACT, anaplastic carcinoma of thyroid; LCA, leukocyte common antigen; UPS, undifferentiated pleomorphic sarcoma.

In Table 2, we reviewed 20 cases of thyroid UPS published in the literature (using Endnote X9 with keywords in PubMed: “malignant fibrous histiocytoma”, “undifferentiated pleomorphic sarcoma”, “the thyroid”). More than 80% of cases were female with a peak of age over 60 years. The thyroid mass in previously reported cases and in our patient exceeded 4 cm in size (75%). The incidence of thyroid cancer within a nodule of greater than 4 cm is 22%^12^; consequently, we could not exclude malignancy even although ultrasound features are benign.

**Table 2:** Review of malignant fibrous histiocytoma/ undifferentiated pleomorphic sarcoma of the thyroid in the literature

Reference, year	Case	Gender/age	Site	Size, cm	Related thyroid disease	Therapy	Survival, months
Zahradka et al, 1989 13	1	M/46	Unknown	Unknown	Unknown	Unknown	4
Malandrinou et al, 2002 14	2	F/42	R	2.5 × 1.6	Thyroid nodule for 5 years	CE	120
Yavuz et al, 2005 15	3	F/60	Unknown	8.0 × 6.0	Increasing thyroid enlargement for 3 years	Unknown	4
Hsu et al, 2008 16	4	F/67	R	5.0 × 2.0	None	CE + 60 Gy	6+
	5	F/70	R	3.5 × 1.2	Basedow for 5 years	CE + 60 Gy	6+
Zeng et al, 2009 17	6	F/66	R	8.0 × 12.0	Two of them have thyroid nodule.	IE + 60 Gy	6
	7	F/54	L	6.0 × 7.0		IE + 70 Gy	10
	8	M/66	R	6.0 × 3.0		CE	24
	9	F/57	R	6.0 × 5.0		IE + C/T	6
	10	F/50	R	2.5 × 1.5		CE	6
	11	F/53	L	5.0 × 6.0		IE + 60 Gy	18
	12	M/57	R	5.0 × 8.0		Biopsy + C/T	Unknown
	13	M/65	R	7.0 × 8.0		IE	14
	14	F/53	R‐L	5.0 × 3.0		IE	3
	15	F/55	L	6.0 × 5.0		30 GY + IE	1
	16	F/47	R‐L	15.0 × 9.0		IE + 60 Gy	4
	17	F/59	R	3.0 × 2.0		CE + 70 Gy	16+
Wang, 2017 18	18	F/78	R‐L	4.6 × 3.6 × 2.9	Thyroid nodule	CE	4
	19	F/68	L	4.3 × 3.4 × 4	Thyroid adenoma	CE	2
Chen, 2018 19	20	F/71	R	5.6 × 4.7.4.5	No	CE + 60 Gy/30 fr, 2 Gy/fr	6+
Our patient	21	F/71	R‐L	4.5 × 3.2	No	CE	6+

Abbreviations: C/T, chemotherapy; CE, complete excision; F, female; IE, incomplete excision; L: left lobe; M, male; R, right lobe.

Complete excision of the primary lesion and involved adjacent tissue is recommended in patients with thyroid UPS in order to achieve negative surgical margins. Radio‐ or chemotherapy is usually indicated as a part of cancer treatment. Because surgery alone has poor results, adjuvant radio‐ and/or chemotherapy is generally considered in soft tissue sarcoma treatment, despite of being no proven benefit. Adjuvant or neo‐adjuvant radiotherapy in the head and neck has been used to improve disease control for thyroid UPS. However, in eleven out of twenty‐one (11/21) cases in which adjuvant radio‐ and/or chemotherapy was used, no proven benefit was found. Rapid local recurrence or distant metastasis usually leads to death.

Ultrasound‐guided RFA for benign thyroid nodules is a minimally invasive treatment modality that may be an alternative to surgery. In our elderly patient, although all of our available tests (a core needle biopsy was denied) initially showed a benign thyroid nodule, we decided to perform total thyroidectomy instead of RFA because of the high risk of malignancy clinically (rapid growth in elderly patient, exceeded 4 cm in size, lacking core needle biopsy). If symptomatic local recurrence after six months coupled with a poor health condition for surgery again, RFA should be an option of choice.

In conclusion, in clinical practice, primary thyroid UPS presents as a rapidly growing neck tumor in the elderly patient that causes compressive symptoms similar to anaplastic thyroid cancer and thyroid lymphoma. Histopathologic features combined with immunohistochemical stains are required for diagnosis. The treatment of choice for primary thyroid UPS is complete excision with or without postoperative adjuvant radio‐ and/or chemotherapy. Although the benefits of this remain unproven.

## CONFLICT OF INTEREST

None declared.

## AUTHOR CONTRIBUTIONS

VBN: designed this study and wrote the preliminary draft of the manuscript. CVL and VVHN: supervised this project. TDC and JHB: performed pathology evaluation. BTT and JHB: revised the manuscript critically. Conflict of interest relevant to this article was not reported.
